# Effective dose of intranasal remimazolam for preoperative sedation in preschool children: a dose-finding study using Dixon’s up-and-down method

**DOI:** 10.3389/fphar.2024.1372139

**Published:** 2024-03-20

**Authors:** Ming-Jie Ni, Yu-Ting Jin, Qian-Lin Wu, Ning Zhang, Jia-He Tian, Jun Li, Kai-Ming Yuan

**Affiliations:** ^1^ Department of Anesthesiology and Perioperative Medicine, The Second Affiliated Hospital and Yuying Children’s Hospital of Wenzhou Medical University, Key Laboratory of Pediatric Anesthesiology, Ministry of Education, Wenzhou Medical University, Key Laboratory of Anesthesiology of Zhejiang Province, Wenzhou Medical University, Wenzhou, Zhejiang, China; ^2^ Department of Anesthesiology, Second Affiliated Hospital of Zhejiang University School of Medicine, Hangzhou, China

**Keywords:** dose-effect relationship, Dixon’s up-and-down method, isotonic regression, intranasal medication, remimazolam

## Abstract

**Background:**

Most preschool children are distressed during anesthesia induction. While current pharmacological methods are useful, there is a need for further optimization to an “ideal” standard. Remimazolam is an ultra-short-acting benzodiazepine, and intranasal remimazolam for pre-induction sedation may be promising.

**Methods:**

This study included 32 preschool children who underwent short and minor surgery between October 2022 and January 2023. After pretreatment with lidocaine, remimazolam was administered to both nostrils using a mucosal atomizer device. The University of Michigan Sedation Score (UMSS) was assessed for sedation 6, 9, 12, 15, and 20 min after intranasal atomization. We used Dixon’s up-and-down method, and probit and isotonic regressions to determine the 50% effective dose (ED_50_) and 95% effective dose (ED_95_) of intranasal remimazolam for pre-induction sedation. Results: Twenty-nine pediatric patients were included in the final analysis. The ED_50_ and ED_95_ of intranasal remimazolam for successful pre-induction sedation, when processed via probit analysis, were 0.65 (95% confidence interval [CI], 0.59–0.71) and 0.78 mg/kg (95% CI, 0.72–1.07), respectively. In contrast, when processed by isotonic regression, they were 0.65 (95% CI: 0.58–0.72 mg/kg) and 0.78 mg/kg (95% CI: 0.69–1.08 mg/kg), respectively. At 6 min after intranasal remimazolam treatment, 81.2% (13/16) of “positive” participants were successfully sedated with a UMSS ≧ 1. All the “positive” participants were successfully sedated within 9 min.

**Conclusion:**

Intranasal remimazolam is feasible for preschool children with a short onset time. For successful pre-induction sedation, the ED_50_ and ED_95_ of intranasal remimazolam were 0.65 and 0.78 mg/kg, respectively.

## 1 Introduction

It has been estimated that up to 75% of children have preoperative anxiety before surgical and diagnostic procedures, which is attributed to factors such as parental separation, needle puncture, uncertainty of medical care, and masked strangers. Immediately before or during the induction of anesthesia, some children may be unable to cooperate and some may protest, fight, or try to escape, which may prolong the induction and be emotionally traumatic for the child (Manyande et al., 2015). Preoperative anxiety may be short, but the consequence of this emotional or psychological stress can be profound and extend to later in life, involving enuresis, somnipathy, and postoperative negative behavioral changes (Watson and Visram, 2003; Batuman et al., 2016). The need to eliminate or minimize children’s anxiety levels before induction has become a consensus among anesthesiologists.

Nonpharmacological or pharmacological efforts have been made to address this issue. Nonpharmacological methods include parental presence at the induction of anesthesia (PPIA), virtual reality (Jung et al., 2021), child life preparation (West et al., 2020), interaction with tablet devices (Kim et al., 2019), and transport in a toy car (Liu et al., 2018; Pastene et al., 2023), but the anxiolytic effectiveness of some is still controversial (Liu et al., 2018; Pastene et al., 2023). Although nonpharmacological means may relieve preoperative anxiety to a certain extent, most are not likely to improve pediatric induction compliance (Jung et al., 2021). Pharmacological agents are more efficient at providing sedation and promoting smooth induction. The ideal pre-induction sedation should be noninvasive, practicable, and controllable, with a rapid onset of action. Oral, intranasal, and rectum administrations of sedative medications are noninvasive methods that both anesthetists and children have accepted well. Oral midazolam is the most popular method for pre-induction sedation. Due to the first-pass effect, the bioavailability of oral midazolam is relatively lower with large variability (Funk et al., 2000; van Groen et al., 2021), and the onset time is as long as 21.9 ± 5.34 min (Lammers et al., 2002). Intranasal medication, instead of the oral route, not only can avoid first-pass metabolism but also offers a rapid onset of therapeutic effects (Bitter et al., 2011) for the rich vascular plexus cavity, which communicates with the subarachnoid space via the olfactory or trigeminal nerve (Trevino et al., 2020). Oral midazolam, intranasal dexmedetomidine, and midazolam (Shen et al., 2022) are practicable, but they are not ideal for pre-induction sedation because of their slow onset or large variability (Wermeling et al., 2006; Baldwa et al., 2012; Nie et al., 2023).

Remimazolam is a water-soluble and ester-based benzodiazepine that is rapidly and extensively metabolized by tissue esterase, with a short elimination half-life (Rogers and McDowell, 2010). The bioavailability of intranasal remimazolam is approximately 50%, which has been validated in adult volunteers (Pesic et al., 2020a). Therefore, it may be promising for pre-induction sedation for pediatric patients. This study aimed to explore the effective dose of intranasal remimazolam for preoperative sedation in preschool children using Dixon’s up-and-down method.

## 2 Methods

### 2.1 Study design and patients

This was an effective dose-finding study based on Dixon’s up-and-down method. The study was reviewed and approved by the Medical Ethics Committee of the Second Affiliated Hospital and Yuying Children’s Hospital of Wenzhou Medical University (Number: 2021-K-118–02) and registered at the Chinese Clinical Trial Registry (chictr.org.cn; registration number: ChiCTR2200059493) before recruitment of the first pediatric patient. Written informed consent was obtained from children’s parents, and children were encouraged to participate in the study. As a result, 32 preschool children were enrolled in the study between October 2022 and January 2023.

### 2.2 Criteria for inclusion and exclusion

Pediatric patients were included if they met the following criteria: age between 3 and 6 years; body mass index (BMI) and body weight were within the normal ranges for the corresponding age; underwent elective circumcision, tonsillectomy, and/or adenoidectomy surgery under general anesthesia; the American Society of Anesthesiologists (ASA) physical status I; and operation time of less than 1 h.

The following exclusion criteria were implemented in this study: upper respiratory tract infection; excessive or obvious runny nose or nasal secretions; known allergy to benzodiazepines; symptomatic cardiovascular or pulmonary diseases; and severe hepatic and kidney function problems. Children who failed nasal medication administration because of low compliance or other reasons were excluded after enrollment.

### 2.3 Pre-induction sedation and anesthesia management

Minimal pre-anesthesia fasting times for all the individuals followed the routine protocol of our hospital, which included clear liquids for 2 h, milk or light foods for 6 h, and solid foods for 8 h. Baseline characteristic data of the candidate children were collected before sedation, including age, gender, weight, height, and ASA status. The modified Yale Preoperative Anxiety Scale (mYPAS) was used to assess preoperative anxiety. The children’s vital indices, including non-invasive blood pressure (NIBP), heart rate (HR), and blood oxygen saturation (SPO_2_), were monitored at 5-min intervals. Afterward, they were sedated in the presence of their parents in the induction room in the waiting area.

To optimize intranasal medication delivery, we used the method of intranasal administration following previous experience (Fantacci et al., 2018), with the final volume close to the ideal volume of 0.2–0.3 mL per nare (Del Pizzo and Callahan, 2014). For this, remimazolam benzenesulfonate powder (Yichang Renfu Pharmaceutical Group Co. Ltd., Yichang, Hubei, China) was diluted in high concentration to minimize the final volume. The final concentration was 50 mg/mL, prepared with 25 mg dissolved in 1 mL of 0.9% normal saline. Both nostrils were used to double the absorptive mucosal surface, using a mucosal atomizer device (MAD) (NSM01, Wuxi Nest Life Technology Co. Ltd., Wuxi, Jiangsu, China) to enhance medication absorption. The MAD is equipped with a soft, conical plug on the tip that forms a seal with the nostril, preventing the expulsion of fluid. When connected with a 1-mL syringe according to the manufacturer’s instructions, the spray atomizes drugs into a fine mist of particles 10–70 microns in size. To minimize the possible irritancy of intranasal remimazolam, we pretreated the nasal cavity with 2% lidocaine-soaked cotton for 3 min. Children sat on their parents’ legs while lying with their heads in the back position, and intranasal remimazolam was rapidly administered toward the top of the ear on the same side by using the MAD. Half the total dose was administered on each side of the nasal cavity within 5 s.

The University of Michigan Sedation Scores (UMSSs) of the children were assessed at 6, 9, 12, 15, and 20 min after intranasal atomization. Then, separation anxiety was evaluated by using the Parental Separation Anxiety Scale (PSAS), and pediatric patients were transferred to the operating room for anesthesia induction; after this, mask acceptance was observed. Nonpharmacological or pharmacological methods were allowed for any difficulties in parental separation if necessary.

Before induction, the children were monitored routinely, as mentioned above. General anesthesia was induced with inhalation of sevoflurane, followed by the establishment of intravenous access. Then, propofol, fentanyl, and a muscle relaxant, if necessary, were administered via intravenous access. Tracheal intubation for tonsillectomy and/or adenoidectomy or laryngeal mask airway for circumcision was selected for ventilation management. Anesthesia was maintained using sevoflurane combined with fentanyl for tonsillectomy and/or adenoidectomy or combined with a penile block for circumcision.

### 2.4 Dixon’s up-and-down method

We used the modified Dixon’s up-and-down method (Pace and Stylianou, 2007) to explore the effective dose of intranasal remimazolam for preoperative sedation in preschool children. Briefly, the initial dose of intranasal remimazolam administered to the first pediatric patient was 1 mg/kg, and the fixed incremental or decremental dose was 0.1 mg/kg, which was 10% of the initial dose. If the former patient was observed to have a negative response, they would be treated with a dose of remimazolam increased by 0.1 mg/kg. Conversely, if a positive response was observed, the next patient received a lower dose with a 0.1 mg/kg decrement. A UMSS maintained at 0 in 20 min was regarded as a negative response to pre-induction sedation.

### 2.5 Outcome assessments

The primary outcome of the current study was the dose of intranasal remimazolam determined for each pediatric patient using Dixon’s up-and-down method.

Secondary outcomes: UMSS at baseline, 6, 9, 12, 15, and 20 min after intranasal atomization; baseline mYPAS score (Kuhlmann et al., 2019); PSAS score (Manoj et al., 2017) 20 min after intranasal atomization; and any adverse effects before anesthesia induction, such as paradoxical reaction, nasal irritation reported by pediatric patients, a desaturation with SPO_2_ less than 92% without oxygen therapy, HR, or NIBP changes of 20% or more.

### 2.6 Statistical analysis

This study was a modified Dixon’s up-and-down sequential allocation trial. Determination of the sample size for Dixon’s up-and-down method is not affected by different experimental types. Typically, without calculating the exact sample size, at least 20–40 patients or subjects are required, and the study must sequentially include them until the conditions of a stopping rule are satisfied (Pace and Stylianou, 2007). This method requires at least six crossover points (non-responsive to responsive) for statistical analysis.

The statistical analysis was performed using SPSS version 16.0 software (SPSS Inc., Chicago, IL, United States). Continuous variables with normal distribution are presented as means (standard deviation), and those with skewed distribution are presented as medians [interquartile range]. For categorical variables, data are expressed as the number (percentage) of patients. The normality of continuous data was assessed using the Shapiro–Wilk test. After obtaining seven “negative–positive” crossover points, probit regression and isotonic regression (Pace and Stylianou, 2007) were also used to process the data from Dixon’s up-and-down method. Then, we derived the 50% effective dose (ED_50_) and 95% effective dose (ED_95_), along with the 95% confidence interval (CI). A *p*-value < 0.05 was defined as statistically significant.

## 3 Results

**FIGURE 1:**
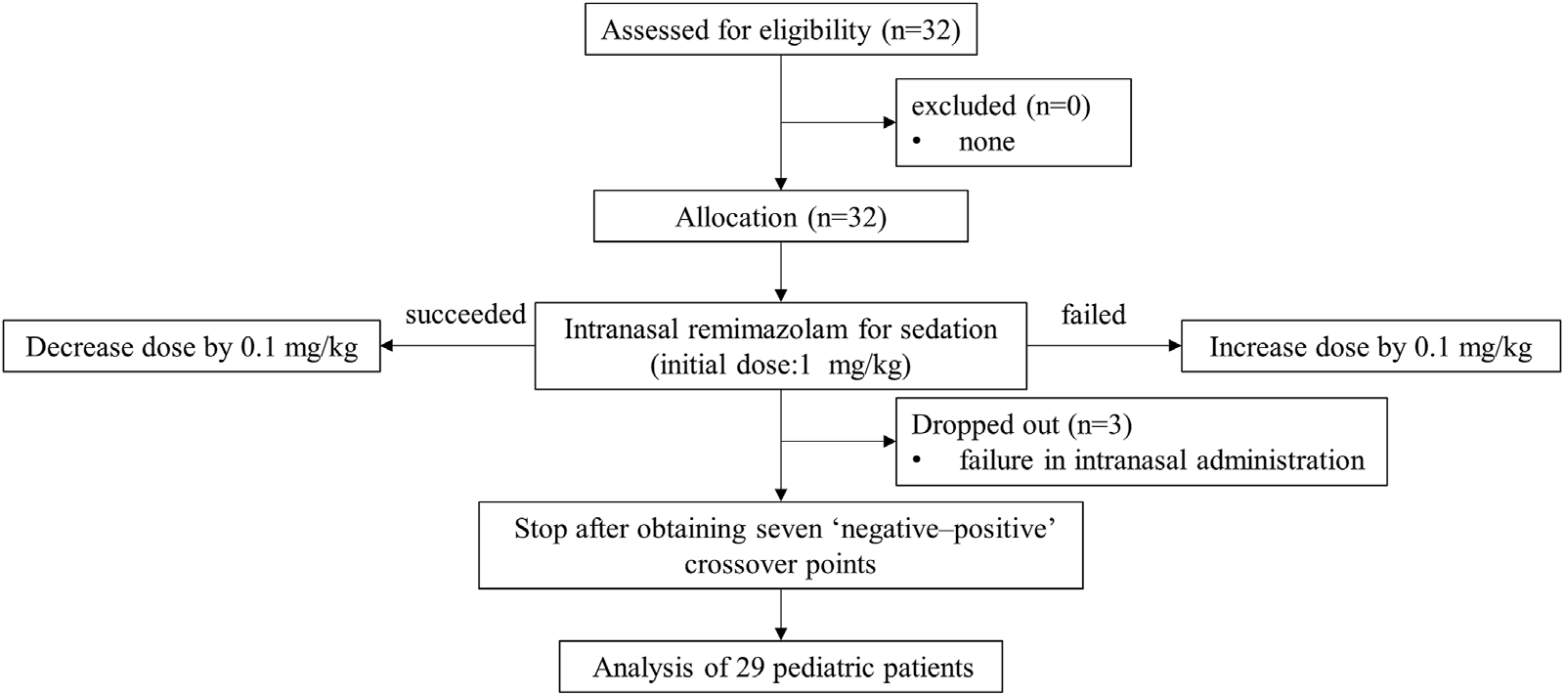
Consort flow diagram of the study.

A total of 32 pediatric patients participated in this study. Three dropped out because of the failure of intranasal administration. Finally, we included 29 patients in the analysis based on Dixon’s method (Figure 1). Table 1 presents the baseline characteristics, with 16 cases of positive responses and 13 cases of negative responses.

**TABLE 1:** Baseline characteristics of the participants.

	Positive (n = 16)	Negative (n = 13)
Gender (M/F)	7/9	5/8
Age (years)	4.8 ± 1.1	4.5 ± 0.8
Weight (kg)	17.9 ± 3.5	16.8 ± 1.6
BMI (kg/m^2^)	15.0 ± 1.6	14.6 ± 1.1
Heart rate (bpm)	102.1 ± 8.3	103.4 ± 11.5
SpO_2_ (%)	100 (1)	100 (1)
mYPAS^a^	43.3 (31.7)	41.7 (23.3)
Operation duration (min)	15.5 (7.5)	14.0 (4.0)
Anesthesia duration (min)	34.8 ± 10.3	35.6 ± 9.5
Type of surgery		
Tonsillectomy and/or adenoidectomy	14	9
Circumcision	2	4

^a^ mYPAS: the modified Yale Preoperative Anxiety Scale.

**FIGURE 2:**
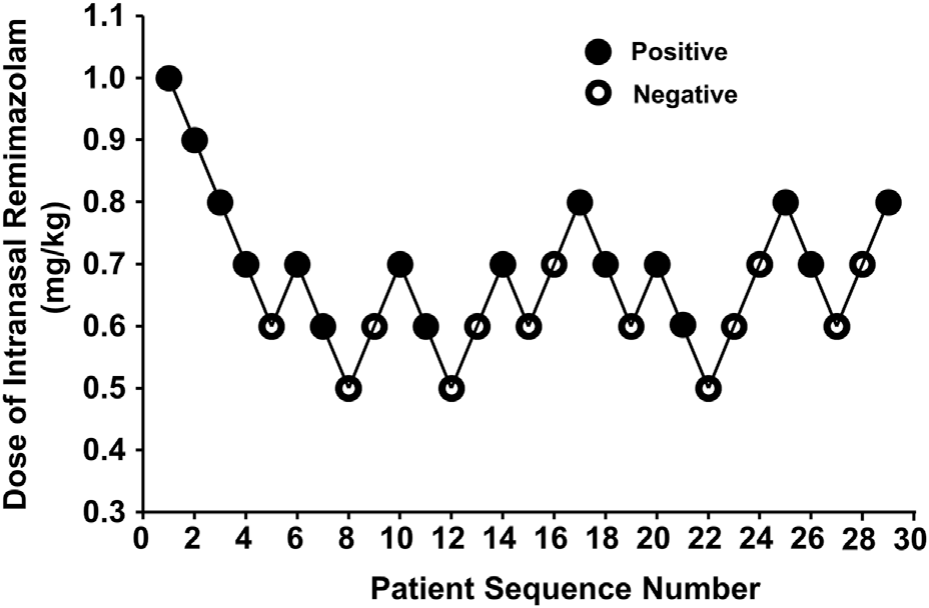
Data for consecutive pre-induction over predetermined doses of intranasal remimazolam (with the initial remimazolam at 1 mg/kg for the first patient). Seven pairs of negative to positive response sequences were used for statistical analysis with Dixon’s up-and-down method. The black dots represent “positive” responses and the white dots represent “negative” responses.

Our study was conducted until data for seven crossover points were collected. Figure 2 shows the sequential response of the 29 pediatric patients to the up-and-down method of intranasal remimazolam. At 6 min after intranasal remimazolam treatment, 81.2% (13/16) of “positive” participants were successfully sedated with UMSSs ≧ 1. All the “positive” participants were successfully sedated within 9 min. One recovered 20 min after treatment. In that case, the dosage was 0.6 mg/kg, which achieved a maximal UMSS of 2 at 12 min. The maximal UMSS observed for each “positive” participant was 2. Thus, all the “positive” participants were moderately sedated. At 12 min after treatment, 13 of 16 “positive” participants achieved maximal UMSSs of 2. PSAS was used to evaluate parent separation anxiety. Only one of the “positive” participants mentioned above who recovered from sedation experienced separation anxiety (PSAS ≧ 1), whereas 6/13 of the “negative” participants experienced separation anxiety. In addition, mask acceptance for induction was excellent in 81.2% (13/16) of participants classified as “positive,” while it was only excellent in 7.7% (1/13) of participants classified as “negative.”

**FIGURE 3:**
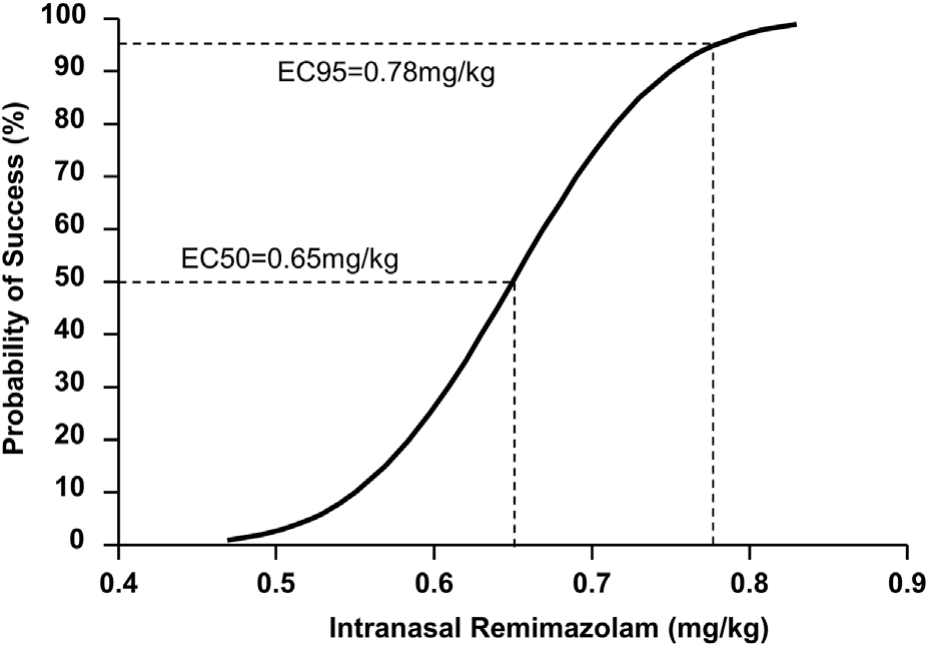
Dose–response curve for pediatric sedation with intranasal remimazolam plotted using probit analysis.

According to the probit analysis, the ED_50_ and ED_95_ of intranasal remimazolam for successful pre-induction sedation were 0.65 (95% CI: 0.59–0.71 mg/kg) and 0.78 mg/kg (95% CI: 0.72–1.07 mg/kg), respectively, as shown in Figure 3.

**FIGURE 4:**
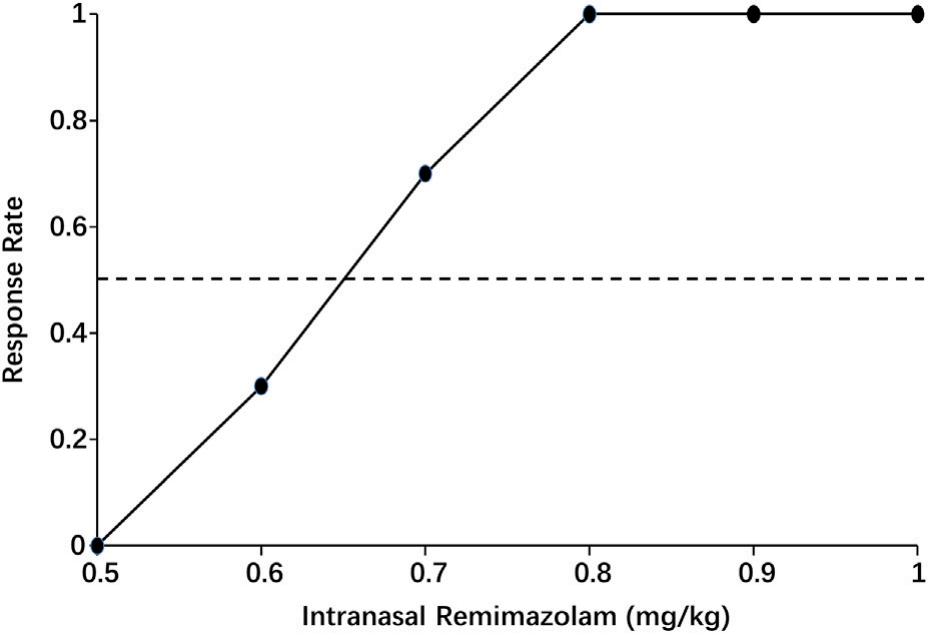
Pooled-adjacent-violators algorithm (PAVA) of the response rate calculated by isotonic regression. The black dots represent the response probability of each dose of intranasal remimazolam. The dashed line represents a response rate of 0.5.

When the data were processed by isotonic regression, the ED_50_ and ED_95_ of intranasal remimazolam for successful pre-induction sedation were 0.65 (95% CI: 0.58–0.72 mg/kg) and 0.78 mg/kg (95% CI: 0.69–1.08 mg/kg), respectively, as shown in Figure 4.

Except for in one participant, there appeared to be paradoxical reactions, with hyperactivity and excitement. No more adverse effects were observed before the induction of anesthesia. No participants reported burning or pain after intranasal administration of remimazolam.

## 4 Discussion

The primary objective of this study was to evaluate the effective dose of intranasal administration of remimazolam to achieve mild to moderate sedation before anesthesia induction in preschool patients. We found that the ED_50_ and ED_95_ of intranasal remimazolam for successful pre-induction sedation, regardless of the regression model used, were 0.65 and 0.78 mg/kg, respectively.

Many children visit the hospital in China for short and minor surgery during winter and summer vacations. A scheme for rapid pediatric sedation without the effect of delayed recovery can improve anesthesia efficiency and accelerate patient turnover. Midazolam is the most popular drug for pediatric sedation. Intranasal midazolam has been used for over 20 years. Studies have shown that, compared with oral midazolam, intranasal midazolam achieved a similar effect in terms of mask acceptance for induction and satisfactory sedation at parental separation, with a shorter onset time (Chen et al., 2023). However, intranasal midazolam does not work fast enough, and, as Heard et al. reported, the onset time is approximately 17 min (Heard et al., 2010). Furthermore, midazolam may delay emergence from general anesthesia in pediatric ambulatory operations (Viitanen et al., 1999).

Remimazolam has a carboxylic ester moiety incorporated into the benzodiazepine core. Thus, it is suitable for rapid metabolism by non-specific tissue esterases in the blood (Rogers and McDowell, 2010). It can produce a rapid onset of action and a short duration of action. Its onset time is only one-third to half that of midazolam after intravenous administration (Borkett et al., 2015). Unfortunately, unlike midazolam, remimazolam cannot be administered orally because of its extremely low bioavailability and distinct bitter taste (Pesic et al., 2020b). However, intranasal remimazolam is feasible, as verified in adult volunteers by Schrier et al. (Pesic et al., 2020a). According to their results, its bioactivity is approximately 50%, and the Cmax was reached after approximately 10 min, which generated an onset within 5 min of administration and achieved peak drowsiness and relaxation within 10–20 min. This is consistent with our present study. We found that at the first time point, 6 min after intranasal administration of remimazolam, 81.2% of the successfully sedated children achieved a UMSS score of 1 or 2. However, at 9 min, 100% of the successfully sedated children achieved a UMSS score of 1 or 2. At 12 min after treatment, 13 of 16 “positive” participants achieved a maximal UMSS of 2. Thus, the onset time of intranasal remimazolam for most children is less than 6 min, and the sedative score peaked at approximately 12 min. Further work should be conducted to explore the onset time of intranasal remimazolam. Because all participants needed to complete 20 min of observation, induction of anesthesia was not performed for some participants at the deepest sedative status. Nevertheless, mask acceptance for induction was excellent in 81.2% of “positive” participants.

Generally, Dixon’s up-and-down methodology requires that the starting dose be the minimum dose expected to result in a positive response. Because the appropriate dose of intranasal remimazolam for pediatric sedation was unknown, we set the remimazolam dose for the first patient at 1 mg/kg. The starting dose per unit weight might be relatively higher than that studied in adults by Schrier et al. (Pesic et al., 2020a). Because water-soluble drugs have larger volumes of distribution in preschool children for the relatively larger amount of body water, the dose of remimazolam for pediatric patients may be larger.

Like midazolam (Chen et al., 2023), intranasal remimazolam causes a high incidence of nasal irritation (Pesic et al., 2020a). Intranasal topical lidocaine pretreatment helps reduce the discomfort associated with intranasal midazolam administration (Smith et al., 2017). In our study, no participants reported sensations of nasal pain, irritation, or burning due to the pretreatment of intranasal topical lidocaine. One of the participants appeared to have symptoms of paradoxical reactions, with hyperactivity and excitement. Although paradoxical reactions are not a rare complication of benzodiazepines, this side effect that occurred in remimazolam needs further assessment. In this study, adverse events, such as respiratory depression, upper airway obstruction, desaturation, or hemodynamic disorders, were not observed before induction of anesthesia. This study did not analyze the side effects after induction due to many confounding factors in small-scale exploratory trials.

For data analysis, probit and isotonic regressions were performed to generate the ED_50_ and ED_95_ of intranasal remimazolam for successful pre-induction sedation. The parameter estimate of the probit regression slope may be biased, and the CIs of the ED_50_ may be unrealistically narrow. We used both probit regression and isotonic models to process the data. The results showed that 95% of ED50 and ED95 were slightly wider under the isotonic regression model. Probit regression is based on the assumption of a normal distribution, while isotonic regression does not involve specific assumptions about the distribution of data. Isotonic regression has been recommended as more appropriate for processing up-down datasets (Pace and Stylianou, 2007). However, the individualized effect of the dose of intranasal remimazolam for children must be widely validated in clinical practice.

Some limitations of our study should be noted. First, our study population was limited to relatively healthy pediatric participants aged 3–6 years. The reason for choosing this age group is that children in this age range account for a large proportion of pediatric surgeries, and pre-induction sedation is strongly recommended. The effective dosage may vary with age in pediatric patients, which needs further measurements. Second, the minimal time interval between the first UMSS scoring and intranasal medication is 6 min; however, our results indicate that the minimal onset time is less than 6 min. Hence, the exact onset could not be determined in the present study. Third, although mYPAS is a good tool for preoperative anxiety evaluation, due to short intervals between time points, we did not conduct mYPAS scoring at each time point but only assessed it at baseline. Moreover, mYPAS is only for patients who are awake, while it is unsuitable for moderately sedated patients. Fourth, children who underwent adenoidectomy surgery may experience obstruction of the choana and nasopharyngeal. It is unknown if this will affect the absorption of a small amount of intranasal remimazolam. Nevertheless, we included these children because adenoidectomy surgery accounts for a significant portion of pediatric short and minor surgeries. Despite these limitations, our findings could help generate a hypothesis regarding the “ideal” method for pediatric sedation and provide valuable data for future studies.

In conclusion, intranasal remimazolam is feasible for preschool children with a short onset time. For successful pre-induction sedation, the ED_50_ and ED_95_ of intranasal remimazolam were found to be 0.65 and 0.78 mg/kg, respectively.

## Data Availability

The raw data supporting the conclusion of this article will be made available by the authors, without undue reservation.
